# Multipotency and cardiomyogenic potential of human adipose-derived stem cells from epicardium, pericardium, and omentum

**DOI:** 10.1186/s13287-016-0343-y

**Published:** 2016-06-13

**Authors:** Wojciech Wystrychowski, Bhagat Patlolla, Yan Zhuge, Evgenios Neofytou, Robert C. Robbins, Ramin E. Beygui

**Affiliations:** Department of Cardiothoracic Surgery, Stanford University School of Medicine, Falk Cardiovascular Research Center, 300 Pasteur Dr, Stanford, CA 94305 USA

**Keywords:** Adipose-derived stem cells, Mesenchymal stem cells, Reprogramming, Cardiogenesis, Adipose tissue, Epicardium, Pericardium, Cardiomyocytes, Fibroblasts, Alkaline phosphatase

## Abstract

**Background:**

Acute myocardial infarction (MI) leads to an irreversible loss of proper cardiac function. Application of stem cell therapy is an attractive option for MI treatment. Adipose tissue has proven to serve as a rich source of stem cells (ADSCs). Taking into account the different morphogenesis, anatomy, and physiology of adipose tissue, we hypothesized that ADSCs from different adipose tissue depots may exert a diverse multipotency and cardiogenic potential.

**Methods:**

The omental, pericardial, and epicardial adipose tissue samples were obtained from organ donors and patients undergoing heart transplantation at our institution. Human foreskin fibroblasts were used as the control group. Isolated ADSCs were analyzed for adipogenic and osteogenic differentiation capacity and proliferation potential. The immunophenotype and constitutive gene expression of alkaline phosphatase (ALP), GATA4, Nanog, and OCT4 were analyzed. DNA methylation inhibitor 5-azacytidine was exposed to the cells to stimulate the cardiogenesis. Finally, reprogramming towards cardiomyocytes was initiated with exogenous overexpression of seven transcription factors (ESRRG, GATA4, MEF2C, MESP1, MYOCD, TBX5, ZFPM2) previously applied successfully for fibroblast transdifferentiation toward cardiomyocytes. Expression of cardiac troponin T (cTNT) and alpha-actinin (Actn2) was analyzed 3 weeks after initiation of the cardiac differentiation.

**Results:**

The multipotent properties of isolated plastic adherent cells were confirmed with expression of CD29, CD44, CD90, and CD105, as well as successful differentiation toward adipocytes and osteocytes; with the highest osteogenic and adipogenic potential for the epicardial and omental ADSCs, respectively. Epicardial ADSCs demonstrated a lower doubling time as compared with the pericardium and omentum-derived cells. Furthermore, epicardial ADSCs revealed higher constitutive expression of ALP and GATA4. Increased Actn2 and cTNT expression was observed after the transduction of seven reprogramming factors, with the highest expression in the epicardial ADSCs, as compared with the other ADSC subtypes and fibroblasts.

**Conclusions:**

Human epicardial ADSCs revealed a higher cardiomyogenic potential as compared with the pericardial and omental ADSC subtypes as well as the fibroblast counterparts. Epicardial ADSCs may thus serve as the valuable subject for further studies on more effective methods of adult stem cell differentiation toward cardiomyocytes.

## Background

Coronary heart disease affects over 15 million Americans with about 120,000 dying from myocardial infarction (MI), making it the leading cause of morbidity and mortality in the United States and worldwide [1]. The survival rate for US patients hospitalized with MI is approximately 95 % and an essential decline in mortality has been reported during the last decades [2]. Significant improvement in survival is related to progress in emergency medical response and treatment strategies, as well as preventative healthcare. Ischemic cardiac tissue is replaced with a fibrotic scar, which leads to a chronic process of cardiac remodeling resulting in compromised ventricular performance and chronic heart failure. Positive correlation between the size of infarction and mortality has been noted [3, 4]. Consequently, the restoration of heart tissue has been the focus of scientific efforts for years. Bergmann et al. [5] have shown that 50 % of human cardiomyocytes (CMs) are replaced during the lifespan. Recent rodent studies confirm the capacity of postnatal CMs to proliferate, and this process is upscaled in the course of myocardial injury [6, 7]. In humans, the regenerative potential of the heart through proliferation of pre-existing CMs seems to be limited to children and adolescents [8]. Other observations point to residual cardiac progenitor cells (CPC) as the possible source for human CMs in vivo [9]. Nevertheless, these physiological processes are limited and insufficient to achieve *restitutio ad integrum* after MI. Application of stem cells or stem-cell-derived CMs is a possible therapeutic approach for improvement of postischemic cardiac function. This has already been confirmed with multiple observations of better heart pump function and overall outcome in the animal model of ischemic heart disease after human embryonic stem cell (ESC) transplantation [10–12]. Nevertheless, application of pluripotent stem cells is connected with a high risk of teratoma formation, which restricts their clinical utilization [13]. Furthermore, ethical concerns exclude broad clinical application of human ESCs. Alternatively, application of mesenchymal stem cells (MSCs) has shown promising results. A reduction of infarct size and an improvement in ventricular remodeling were observed in patients with ischemic cardiomyopathy after administration of bone marrow-derived MSCs (BM-MSCs) (POSEIDON and REPAIR-AMI studies) [14–16]. Similar or better results were achieved with transplantation of the CPC subsets: cardiosphere-derived cells (CADUCEUS study) and c-kit-positive cardiac stem cells (SCIPIO trial) [17, 18]. Observed amelioration of the cardiac function is caused predominantly by the paracrine anti-inflammatory and antiapoptotic effect, as well as neovascularization with stem cell differentiation into endothelial and smooth muscle cells [19–21]. In addition, transplanted CPCs are supposed to stimulate proliferation of the preexisting CMs and/or cardiogenesis of the residual CPCs. Nevertheless, there is no evidence for the successful cardiac differentiation of transplanted MSCs or CPCs in humans. Strategies based on in-vitro differentiation of the stem cells toward CMs followed by their transplantation into ischemic myocardium were possible with ESCs and induced pluripotent stem cells (iPSCs) only. Nevertheless, the differentiation efficacy remained low, with phenotypical immaturity of the iPSC-derived CMs [22]. Furthermore, arrhythmias were observed in a nonhuman primate model of iPSC-CM transplantation [23]. Different promising strategies are based on direct transdifferentiation of mature somatic cells into CMs, thus omitting the pluripotent state. This approach was applied by Fu et al. [24] who presented a successful direct reprogramming of human fibroblasts toward CMs in vitro. The clinical translation of such a strategy will allow transformation of the cardiac postischemic scar to a functional myocardium.

Diverse differentiation abilities have been observed for stem cells derived from bone marrow and different adipose tissue compartments. Nevertheless, the majority of previous studies on the characteristics of different sources of ADSCs do not discern the epicardial and pericardial adipose tissue. This is influenced by the fact that little or no epicardial fat is present in rodents, which is the most common experimental animal model. Because of close anatomical and physiological connections with the heart, we hypothesized that ADSCs from epicardium and pericardium may express a high cardiomyogenic potential. Nevertheless, both differ in their morphogenesis, anatomy, and physiology, which may influence ADSC differentiation and expansion abilities [25, 26]. Furthermore, proepicardium plays a crucial role in cardiac morphogenesis. During cardiogenesis epicardial cells are subjected to epithelial–mesenchymal transformation, allowing their further migration and differentiation into the cardiac fibroblasts, smooth muscle cells, and putatively CMs [27–29]. Moreover, epicardial fat exerts the origin and characteristics of brown adipose tissue [30]. Experimental trials confirm a higher ability of brown ADSCs to differentiate into CMs, as compared with cells derived from white adipose tissue [31, 32].

Taking into account these observations, we aimed to analyze and compare key morphological features and differentiation abilities, including cardiomyogenic potential, of epicardial (E-ADSCs), pericardial (P-ADSCs), and omental (O-ADSCs) stem cells derived from adipose tissue, as well as human foreskin fibroblasts as the control cell line. We aimed to apply the 5-azacytidine (5-aza)-induced cardiac differentiation method, previously proven for successful BM-MSC cardiogenesis [33]. Furthermore, we tested the ADSC transdifferentiation method, adopting the reprogramming approach described by Fu et al. for fibroblasts [24].

## Methods

### Tissue procurement

Stanford University IRB approved discarded human tissue utilization for research purposes (approval #16440 and #19810). The pericardial and omental adipose tissue samples were harvested from five deceased organ donors, without chronic comorbidities. The ventricular epicardial fat tissue was procured from one organ donor and four explanted hearts from cardiac transplant recipients with comorbidities, including heart failure and atherosclerosis. None of the donors had insulin-dependent diabetes. Average age in all three groups did not differ significantly: 46.9 ± 10.8 (*n* = 5, O-ADSCs and P-ADSCs) and 58.5 ± 9.2 (*n* = 5, E-ADSCs). The human foreskin fibroblast cell line (BJ fibroblasts) was obtained from the American Type Culture Collection (ATCC # CRL-2522).

### ADSC isolation and culture

Harvested tissue (20–50 ml) was washed in sterile PBS (10010; Gibco) containing 1 % penicillin and streptomycin (P/S, P4333; Sigma-Aldrich) and cleaned of any visible blood vessels and fibrotic layers, minced mechanically with sterile scissors, and digested with 0.01 % collagenase IV (17104-019; Life Technologies) DMEM F12 (SH3027101; HyClone) solution for 2 h at 37 °C. The acquired cell suspension was centrifuged at 300 × *g* for 5 minutes. The supernatant was discarded and a stromal vascular fraction pellet was collected, washed, and filtered (100 μm and 40 μm cell strainer). The achieved cell fraction was plated onto cell culture flasks and cultured in low-glucose DMEM media (11885; Gibco) supplemented with 10 % FBS (ES-009-B; Millipore) and 1 % P/S in 5 % CO_2_ at 37 °C. Cultures were washed after 72 h to remove unattached cells and expansion medium was changed every 48 h thereafter. To keep cells at low density, preventing cell death and spontaneous differentiation, cultures were repassaged when reaching 80 % confluence. Cells were harvested using 0.25 % trypsin (TrypLE™; Life Technologies) for 3 min at 37 °C, followed by trypsin inactivation using 2 volumes of culture medium containing serum and replated onto new cell culture dishes/flasks at a seeding density of 3 × 10^4^ per 75 cm^2^ culture flask.

### Osteogenesis and adipogenesis

Each cell line for each adipose tissue sample (passage 5, 5 × E-ADSCs, 5 × P-ADSCs, 5 × O-ADSCs, and 1 × BJ fibroblasts) was seeded on three 12-well plates at a seeding density of 10 × 10^3^/well and cultured to reach 80 % confluence. The control cells were cultured continuously in the expansion medium (low-glucose DMEM (11885; Gibco), 10 % FBS, 1 % P/S). Differentiation media were prepared *ex tempore*, as described previously [34]. Briefly, for osteogenic differentiation, the expansion medium was supplemented with dexamethasone (0.1 μM, D4902; Sigma-Aldrich), β-glycerol phosphate (10 mM, G9891; Sigma-Aldrich), and ascorbic acid (50 μM, A4544; Sigma-Aldrich). Concurrently, adipogenesis was stimulated with expansion medium supplemented with dexamethasone (1 μM, D4902; Sigma-Aldrich), isobutylmethylxanthine (0.5 mM, I5879; Sigma-Aldrich), and indomethacin (200 μM, I7378; Sigma-Aldrich). Respective expansion/differentiation medium was changed every 72 h. Successively, 1, 2, and 3 weeks after induction, the differentiation potential was analyzed and compared. Part of the treated and control cell cultures from each group were fixed with 4 % paraformaldehyde for 10 min, washed three times with PBS, and stored at 4 °C until processing/analyzing. Osteogenic differentiation was evaluated by cellular alkaline phosphatase (ALP) activity (Alkaline Phosphatase kit, 86R; Sigma-Aldrich) and staining for mineralized matrix (Alizarin red S, A5533-25G; Sigma-Aldrich). Staining was quantified by spectrophotometry, measuring the absorbance at 405 nm and 550 nm, for ALP and Alizarin, respectively (Tecan InfiniteM200 plate reader; GENios, Switzerland). Adipogenesis was confirmed by Oil red O staining of intracellular lipids. Quantification was based on counting stained cells.

### Differentiation towards CMs with 5-aza

E-ADSCs, P-ADSCs, and O-ADSCs as well as BJ fibroblasts were plated and cultured in expansion medium (passage 4, low-glucose DMEM (11885; Gibco), 10 % FBS, 1 % P/S at 37 °C in 5 % CO_2_) in four replicates. When the cells reached 80 % confluence, half of the cell cultures were treated with 10 μM 5-aza (Sigma-Aldrich) for 24 h, as described previously [35, 36]. The following day, differentiation medium was removed and washed with sterile PBS, and the cell cultures were continued in expansion medium for another 3 weeks. The cellular morphology was monitored under a microscope every other day. After 21 days of culture, immunohistochemistry and gene expression analysis were performed.

### Direct differentiation of ADSCs into CMs using seven retroviral transcription factors

The retroviral vectors harboring ESRRG, GATA4, MEF2C, MESP1, MYOCD, TBX5, and ZFPM2 (seven factors) and YFP were transfected into HEK293 cells with FuGene 6 (E269; Promega) as described elsewhere [24] (retroviral vectors obtained from Dr Srivastava’s lab, Gladstone Institute, UCSF). Reprogramming medium containing generated virus was collected next. The ADSC subsets and BJ fibroblasts were seeded on 12-well plates (passage 4) in four replicates for each cell line (5 × E-ADSCs, 5 × P-ADSCs, 5 × O-ADSCs, and 1 × BJ fibroblasts) and cultured in an expansion medium until they reached 80 % confluence. Subsequently, the cell transduction with seven factors was performed by 12-h incubation in fresh expansion medium supplemented with reprogramming viral solution (20 μl of each/well of 12-well plate) and polybrene (4 μg/ml) or polybrene only (respective control group). Effectiveness of the cell transduction was assessed by the appearance of YFP-positive cells under fluorescent microscopy. Cell cultures were continued for the following 3 weeks in the CM culture medium (80:20 DMEM, M199 (11150; Gibco), 10 % FBS, P/S, Non-Essential Amino Acids Solution (11140; Gibco)). The culture medium was changed every 48 h. Immunohistochemistry and gene expression analysis was performed 21 days after reprogramming.

### Real-time reverse transcriptase PCR

Total RNA was extracted from collected cells using Trizol reagent (15596; Life Technologies). The cDNA was then synthesized, using SuperScript III First-Strand Synthesis SuperMix (18080-400; Invitrogen). Real-time PCR was performed on TaqMan (Applied Biosystems) in duplicate. MasterMix (Applied Biosystems) and TaqMan probes (Life Technologies) were used as follows: ALP, Hs01029144_m1; GATA4, Hs00171403_m1; Nanog, Hs04260366_g1; OCT4, Hs04260367_gH; cardiac troponin T (cTNT), Hs00943911_m1; and alpha-actinin 2 (Actn2), Hs00153809_m1. The amount of the target gene was normalized to an endogenous reference gene 18S (4333760; Life Technologies). Results were analyzed using the 2^–ΔΔCt^ method and expressed as the fold change in gene expression relative to the respective control groups or BJ fibroblasts.

### Flow cytometry analysis

A single cell suspension of analyzed cells was collected by treating the cells with 0.25 % trypsin for 3 min at 37 °C, followed by washing in complete culture medium. Cells were washed twice with 3 % FCS–PBS (ES-009-B; Millipore), resuspended at a concentration of 5 × 10^5^ cells/ml, and stained with allophycocyanin, fluorescein isothiocyanate, phycoerythrin, Alexa 647, or PerCP/Cy5.5-conjugated monoclonal mouse-anti-human CD29, CD31, CD34, CD44, CD45, CD73, CD90, CD105, and CD166 antibodies and lineage cocktail 1 (lin-1; BD Biosciences: CD3, CD14, CD16, CD19, CD20, CD56) at 4 °C for 30 min. Cells were then washed twice, resuspended, and analyzed for cell surface marker expression by flow cytometry (FACSCalibur™; BD Biosciences).

### Immunocytochemistry

Cultured cells were fixed with 4 % paraformaldehyde for 15 min, washed twice with PBS, and subsequently permeabilized with 0.5 % Triton X-100 (X100; Sigma-Aldrich) for 10 min. The washed cells were blocked for 30 min at room temperature with 1 % BSA (A9418; Sigma-Aldrich), and then incubated overnight at 4 °C with primary monoclonal mouse anti-alpha-actinin (A7811; Sigma-Aldrich) antibody diluted in 0.2 % BSA solution. After three serial washes with PBS, cells were incubated with AlexaFluor 594 conjugated goat anti-mouse antibody for 1 h at room temperature, and then washed and incubated in 300 nM DAPI for 5 min for nuclear counterstain.

### Proliferation assay

For the comparison of cell proliferation, 10 × 10^3^ cells were plated into each well of a 12-well plate and cells were counted daily in triplicate for 14 days. Doubling time (Td) for each cell sample was calculated by the following formula:

$$ \mathrm{T}\mathrm{d} = \Delta t* \ln 2/ \ln \left(\Delta N\right) $$

where Δ*t* is the analysis time period and Δ*N* is the cell number increment. A mean doubling time was calculated for each cell subtype further on.

### Statistical analysis

Statistical analysis was performed with STATISTICA (StatSoft). All variables are expressed as mean ± standard error (SEM). Significant differences between two groups were determined by the independent Student’s *t* test. Analysis of variance (ANOVA) with Fisher post-hoc comparison was performed to evaluate repeated measures. *P* < 0.05 was considered statistically significant.

## Results

### ADSC morphology, genotype, and phenotype

**Fig. 1:**
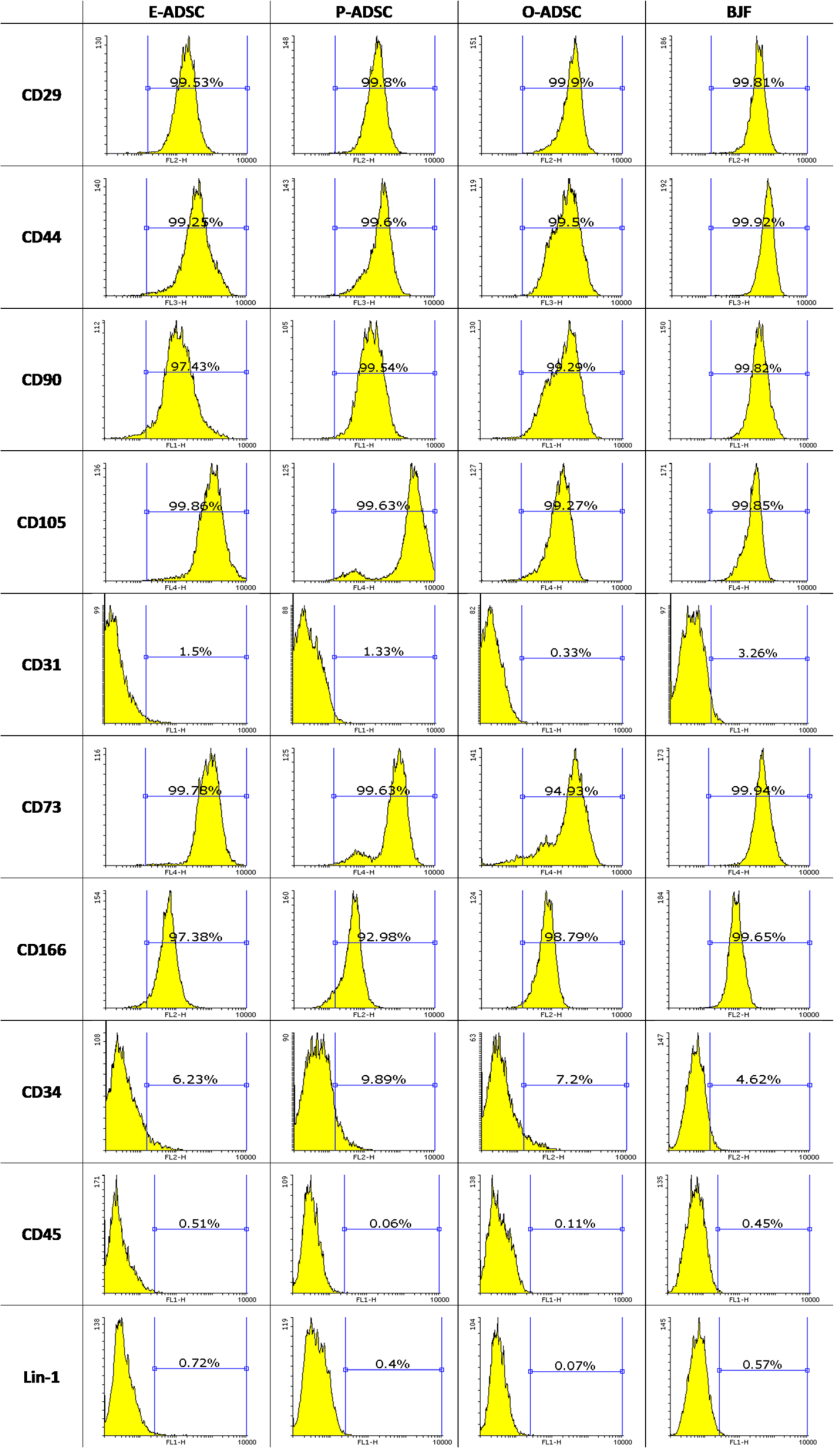
Characterization of human ADSC subsets and BJ fibroblasts (*BJF*). Results of flow cytometric analysis. ADSCs and BJ fibroblasts were positive for the hallmark pattern of MSCs (CD29, CD44, CD90, CD105) and were negative for CD31, CD34, and hematopoietic marker CD45. *E/O/P-ADSC* epicardial/omental/pericardial adipose-derived stem cell

**Fig. 2:**
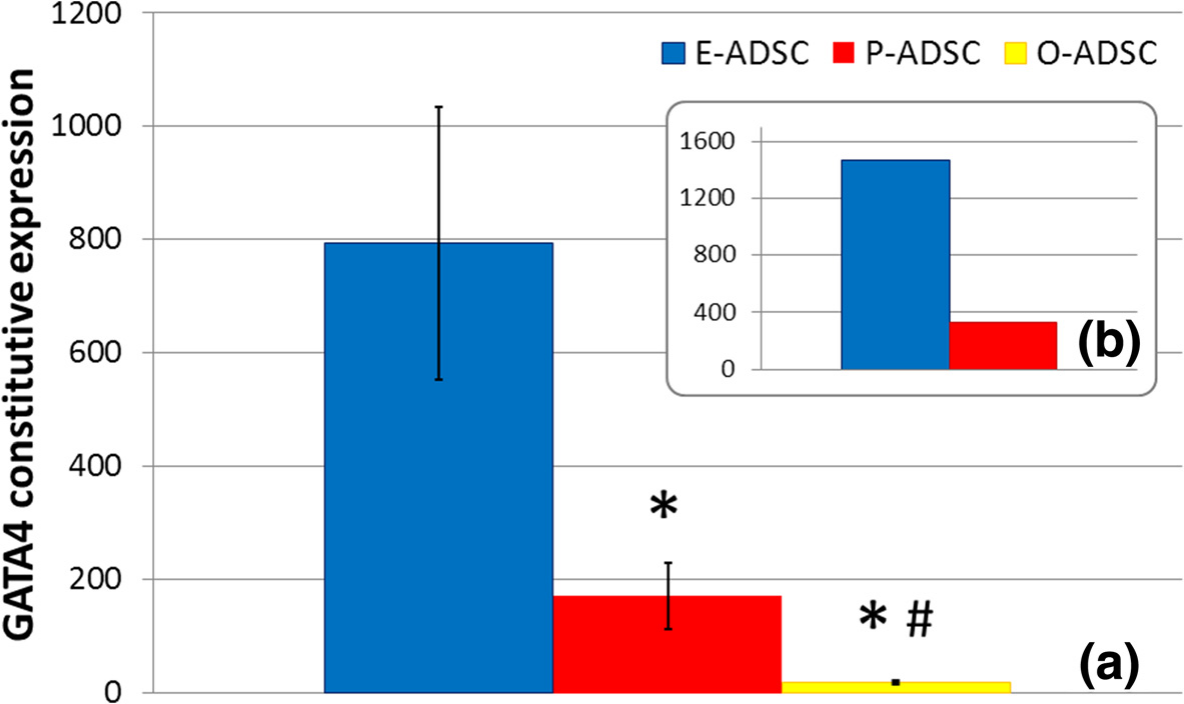
ADSC constitutive expression of the GATA4 gene. Quantitative real-time RT-PCR results performed in duplicate represented by fold-change values as compared with fibroblasts, with a standard 18S endogenous control. Comparison of the collected ADSC subtype samples (*n* = 5 adipose tissue samples for each ADSC subtype, mean ± SEM; *P < 0.05 vs E-ADSCs, #*P* = 0.06 vs P-ADSCs) (**a**), and epicardium and pericardium-derived stem cells harvested from the same brain-dead organ donor free of comorbidities (**b**). *E/O/P-ADSC* epicardial/omental/pericardial adipose-derived stem cell

**Fig. 3:**
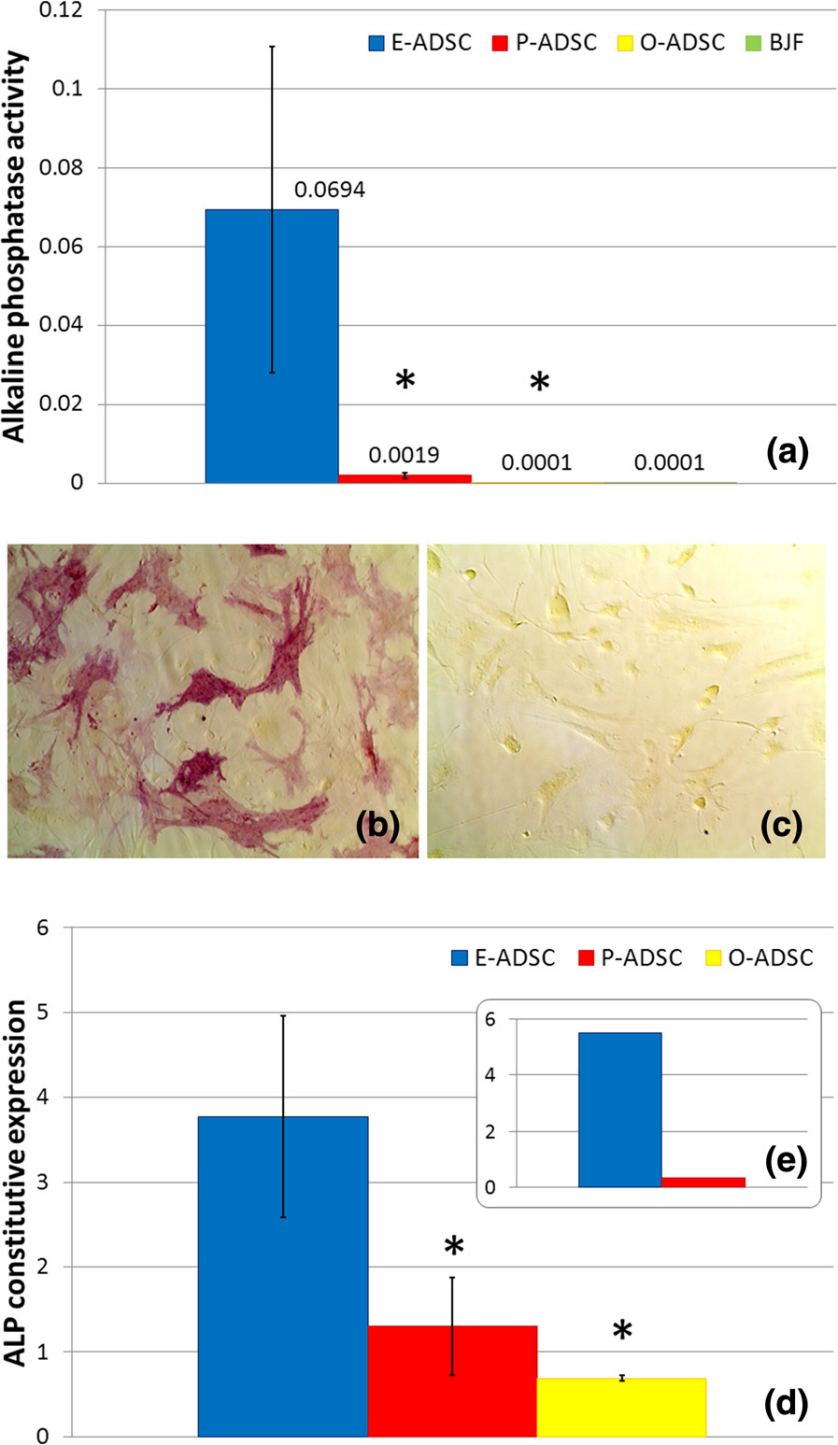
ADSC and BJ fibroblast (*BJF*) ALP activity (**a**–**c**) and constitutive ALP gene expression (**d**, **e**). Comparison of ALP activity quantitated by colorimetric analysis of the constitutive enzyme activity in passage 5 ADSCs and BJ fibroblasts (*n* = 5, mean ± SEM, *P < 0.05 vs E-ADSCs) (**a**). ALP staining in the same donor epicardial (**b**) and pericardial (**c**) ADSCs. Quantitative real-time RT-PCR results performed in duplicate represented by fold-change values as compared with fibroblasts, with a standard 18S endogenous control. Comparison of the collected ADSC subtype samples (*n* = 5 adipose tissue samples for each ADSC subtype, mean ± SEM, *P < 0.05 vs E-ADSCs) (**d**), and epicardium and pericardium-derived stem cells harvested from the same brain-dead organ donor free of comorbidities (**e**). *ALP* alkaline phosphatase, *E/O/P-ADSC* epicardial/omental/pericardial adipose-derived stem cell

**Fig. 4:**
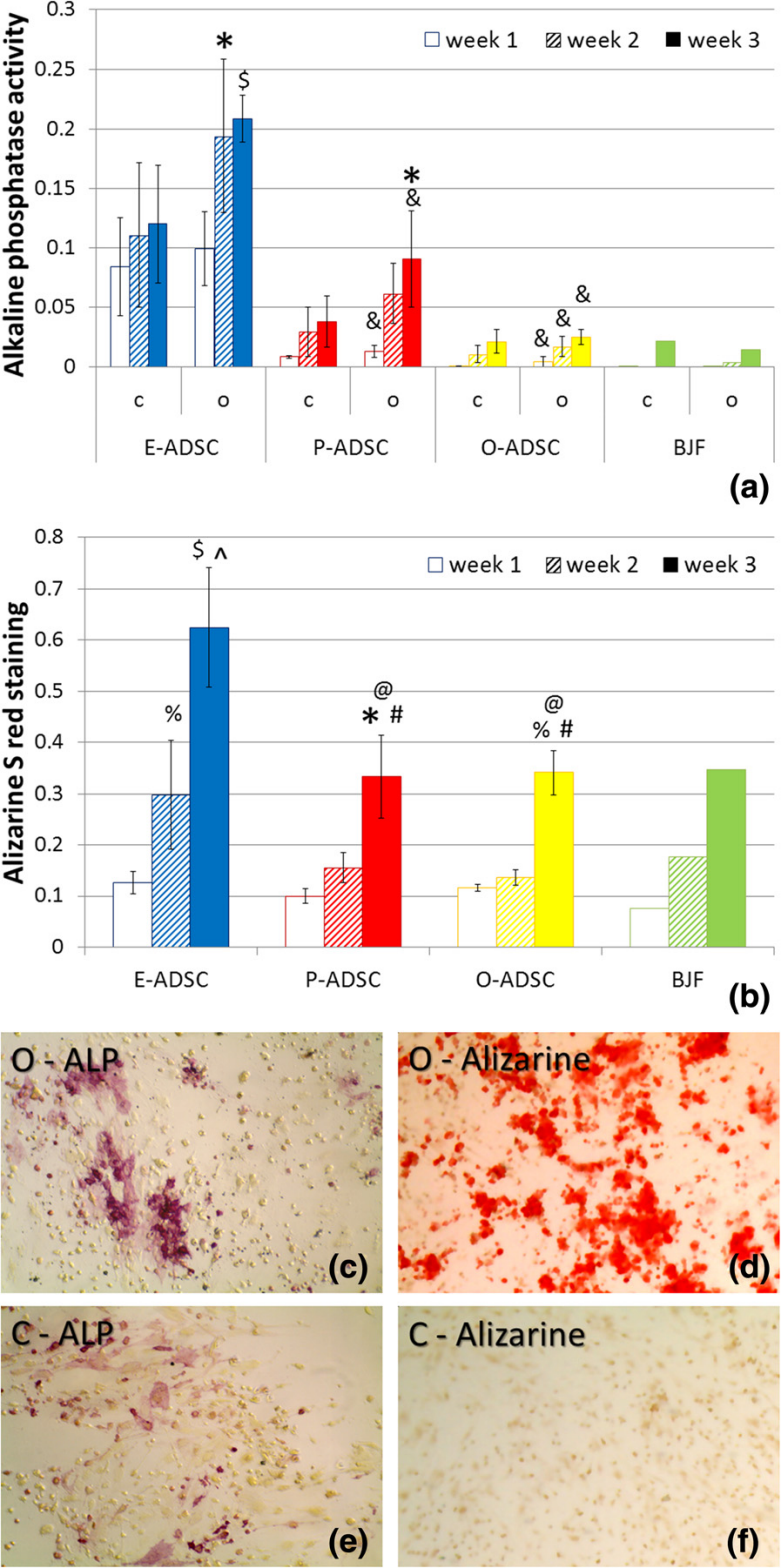
Evaluation of ADSC osteogenic potential. Changes of ALP activity (**a**) and calcium mineral deposition (**b**) over 3 weeks of osteogenic differentiation (*O*), as compared with the respective control culture and between subsets (*C*), quantitated by colorimetric analysis (*n* = 5 adipose tissue samples for each ADSC subtype, mean ± SEM; ^$^ *P* < 0.001, *P < 0.005, ^%^ *P* < 0.05 vs week 1; ^*P* < 0,001; ^#^ *P* < 0.05 vs week 2; and &*P* < 0.05, ^@^ *P* = 0.05 vs respective E-ADSCs). Example of P-ADSC ALP and Alizarin red S staining after 3-week osteogenesis (**c**, **d**) or control culture (**e**, **f**). *ALP* alkaline phosphatase, *E/O/P-ADSC* epicardial/omental/pericardial adipose-derived stem cell

Isolated ADSCs exhibited a fibroblast-like morphology with the property of plastic adherence. Several surface markers were analyzed via flow cytometry. All analyzed ADSC subsets (passage 3–4) were characterized by positive constitutive expression of CD29, CD44, CD90, and CD105 (the hallmark pattern of MSCs), as well as CD73 and CD166, and were negative for the CD31, CD34, and a hematopoietic marker CD45 [37]. BJ fibroblasts expressed a similar phenotype (Fig. 1). Transcription factor GATA4 plays a key role in myocardial morphogenesis [38, 39]. GATA4 overexpression is an essential cofactor in the stem cells and fibroblasts reprogramming toward CMs in vitro [24, 40, 41]. E-ADSCs were characterized by nearly four times higher constitutive expression of GATA4 as compared with P-ADSCs. Both showed higher GATA4 expression than O-ADSCs and fibroblasts (Fig. 2). Nanog and OCT4 are recognized as pluripotency markers, highly expressed in ESCs and iPSCs. The analyzed cell lines did not differ significantly in expression of these genes. Comparison of the same donor samples revealed slightly higher expression of OCT4 and Nanog in E-ADSCs, as compared with P-ADSCs (1.12× and 1.22×, respectively). Interestingly, E-ADSCs were characterized with a high constitutive activity of the ALP (Fig. 3a–c) and ALP gene expression (Fig. 3d,e), as compared with the other ADSC subtypes and BJ fibroblasts. In all analyzed ADSC subsets, ALP activity increased along the length of cell culture (*F* = 4.35, *P* = 0.02; Fig. 4a). Negative Alizarin red S staining excluded spontaneous osteogenic transformation as the cause of the growing presence of ALP-positive cells (Fig. 4c–f).

### ADSC osteogenic and adipogenic differentiation

**Fig. 5:**
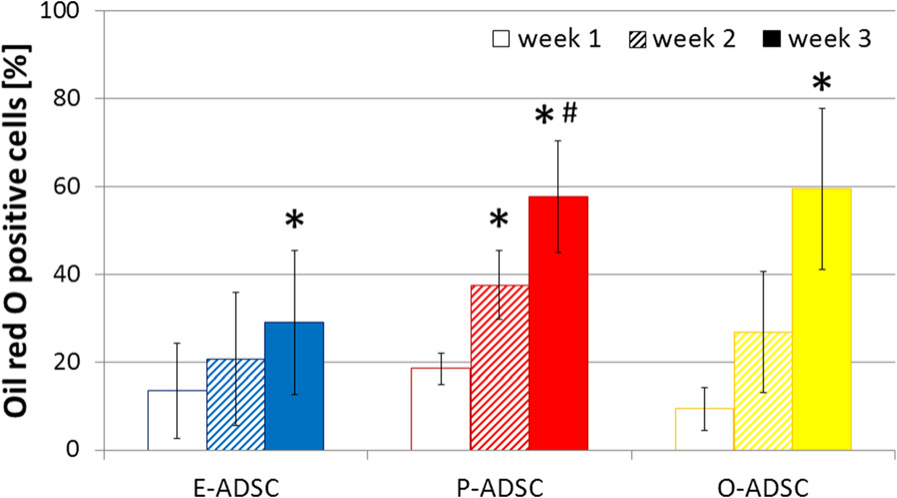
Evaluation of ADSC adipogenic potential. Comparison of adipogenic transformation efficiency between E-ADSCs, P-ADSCs, and O-ADSCs, as determined by Oil red O staining 1, 2, and 3 weeks after differentiation onset (*n* = 5 adipose tissue samples for each ADSC subtype, mean ± SEM, *P < 0.05 vs week 1, ^#^ *P* < 0.05 vs week 2). BJ fibroblasts were negative for Oil red O staining after 3-week adipogenic differentiation. *E/O/P-ADSC* epicardial/omental/pericardial adipose-derived stem cell

All analyzed human ADSC lines revealed multipotent properties with an osteogenic and adipogenic differentiation potential. Extracellular mineralization, confirmed with Alizarin red S staining, was nearly twofold more intense in epicardial than pericardial and omental ADSC cultures, after 2 and 3 weeks of osteogenesis (Fig. 4b). Furthermore, it correlated positively with the respective ADSC constitutive ALP activity (week 2: *R* = 0.86 *P* < 0.05, week 3: *R* = 0.67 *P* < 0.05). As expected, ALP activity increased during osteogenesis in all ADSC subsets (Fig. 4a) and correlated positively with its activity in respective controls (*R* = 0.81, *P* < 0.05). E-ADSCs were characterized with the highest ALP activity increase over 3-week osteogenic differentiation. On the contrary, E-ADSCs revealed lower adipogenic potential as compared with the highly adipogenic O-ADSCs (Fig. 5). These data points suggest a distinct differentiation potential of the epicardial, pericardial, and omental stem cells derived from adipose tissue.

### Proliferation

**Fig. 6:**
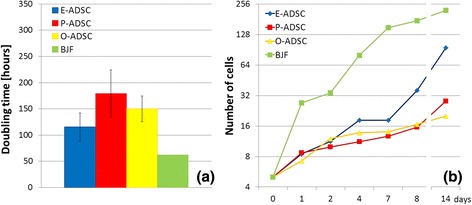
Comparison of proliferation abilities of the analyzed ADSC subtypes and BJ fibroblasts (*BJF*). Cell proliferation expressed as the doubling time (**a**) and the increase of cell number within 2 weeks of cell culture (log2 scale) (**b**). *E/O/P-ADSC* epicardial/omental/pericardial adipose-derived stem cell

We confirmed the exponential long-term growth of all ADSC subtypes with 4 months of continuous culture. E-ADSCs exhibited a higher proliferation potential than pericardium and omentum-derived cells (Fig. 6).

### Cardiomyogenic differentiation

**Fig. 7:**
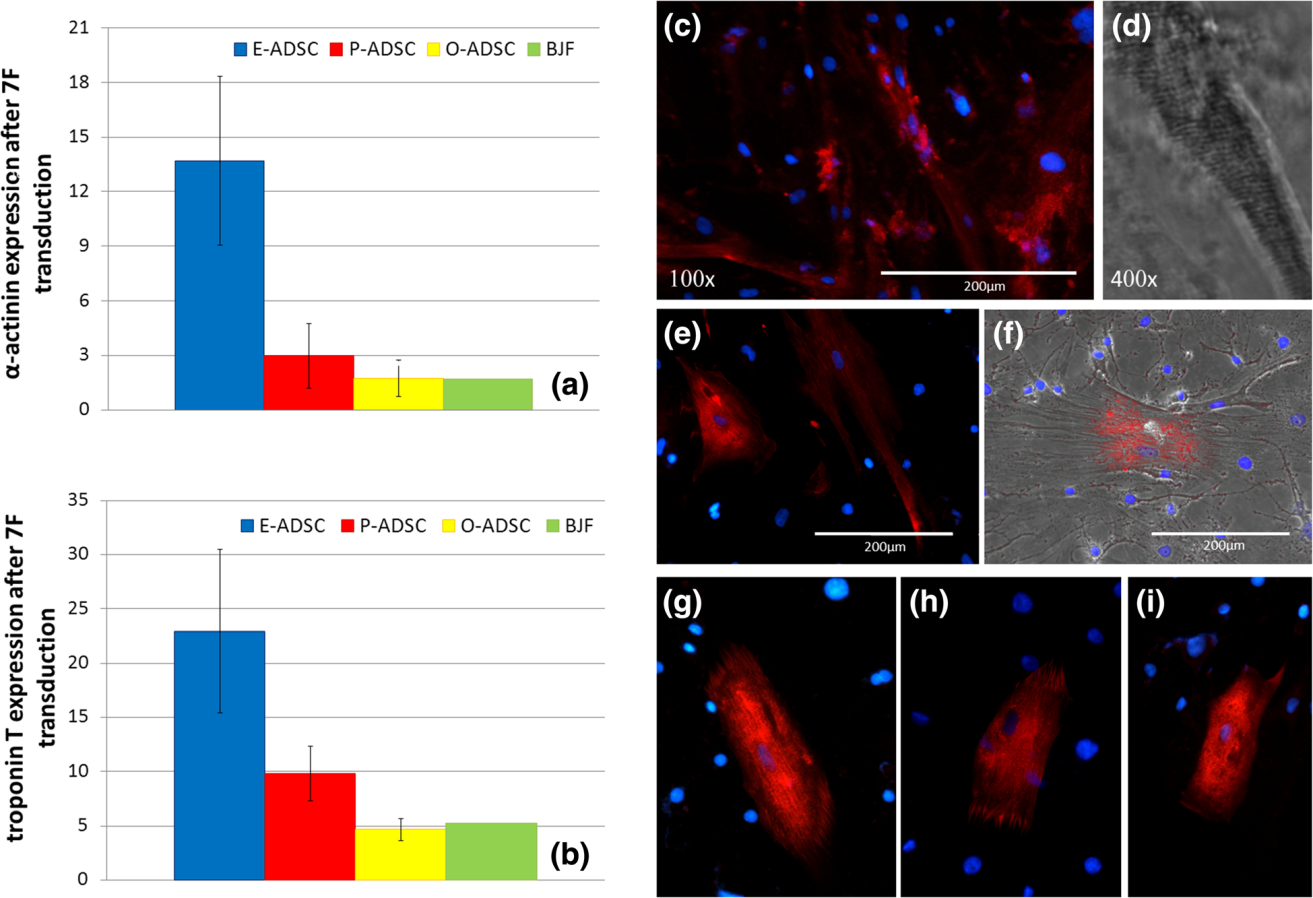
E-ADSCs, P-ADSCs, and O-ADSCs 3 weeks after seven-factor transduction. Expression of Actn2 and cTNT. Quantitative real-time RT-PCR results performed in duplicate represented by fold-change values in the treated culture (reprogramming viral solution + polybrene) as compared with the respective control (polybrene only) (*n* = 5 adipose tissue samples for each ADSC subtype) (**a**, **b**). Bright-field microscopy and anti-alpha-actinin immunostaining (E-ADSCs **c**–**f**, P-ADSCs **g**, **h**, O-ADSCs **i**), revealing alpha-actinin-positive cells, changes in cell morphology (**c**, **e**–**i**), and striations (**d**). *BJF* BJ fibroblasts, *E/O/P-ADSC* epicardial/omental/pericardial adipose-derived stem cell, *7 F* seven factor (GATA4, MEF2C, TBX5, ESRRG, MESP1, MYOCD, ZFPM2)

First, we analyzed the effectiveness of 5-aza-induced cardiogenesis of human ADSCs, as described previously [33]. E-ADSCs, P-ADSCs, and O-ADSCs did not show any cardiac-specific morphological changes 21 days after exposure to 5-aza. Furthermore, they did not stain positively for alpha-actinin and did not reveal increased expression of Actn2 and cTNT, as compared with the respective control culture. Subsequently, we analyzed induction of cardiogenesis with seven factors (GATA4, MEF2C, TBX5, ESRRG, MESP1, MYOCD, ZFPM2) and YFP. As described by Fu et al. [24], these were shown to initiate reprogramming of human cardiac fibroblasts toward CMs in vitro. Three weeks after transduction with the seven factors, E-ADSCs revealed 14 and 22 times higher expression of Actn2 and cTNT, respectively, as compared with the control culture (Fig. 7a,b). Concurrently, at the same transfection effectiveness, their relative expression was, on average, at least twice lower in P-ADSCs, O-ADSCs, and fibroblasts. PCR results were confirmed with immunohistochemistry showing alpha-actinin-positive cells, exhibiting changes to an elongated morphology. Single striated cells were noted in transduced E-ADSCs only (Fig. 7c–i). Interestingly, analyzing all ADSC subsets, Actn2 expression 3 weeks after seven-factor transduction correlated positively with the constitutive GATA4 expression (*R* = 0.79, *P* < 0.05). However this dependence was not observed in the case of E-ADSCs, and consequently the correlation was stronger for P-ADSCs and O-ADSCs (*R* = 0.87, *P* < 0.05).

## Discussion

Chong et al. have shown that perivascular multipotent stem cells, which are present in the epicardial layer, do not originate from the bone marrow despite having the same antigen profile as BM-MSCs. Furthermore, they revealed their potential role in cardiac regeneration after MI [42]. Here we have confirmed the presence of human MSCs in both pericardial and epicardial adipose tissue, presenting dominant but distinct multipotent characteristics. Both thoracic lineages revealed a higher constitutive ALP expression and activity, and lower adipogenic potential, as compared with O-ADSCs. This result is consistent with observation of the reciprocally competing osteogenic and adipogenic capacity of MSCs [43]. Previous studies disclosed the presence of ALP-positive human BM-MSCs in young patients [44]. D’Ippolito et al. [45] described ALP^+^ BM-MSCs as the osteoprogenitors and similarly observed their declining number in aging patients. Simultaneously, a few observations point to the higher proliferation potential of MSCs/ADSCs in juveniles [46]. Our analysis confirmed a positive correlation between constitutive ALP expression and ADSC osteogenic potential. These results would seem consistent with previous observations unless we observed the highest ALP expression in the epicardium-derived stem cells, physiologically unrelated to neo-osteogenesis in vivo. Interestingly, apart from the higher ALP expression, E-ADSCs were characterized by higher proliferation potential. In line with these observations, we hypothesize that ALP-positive ADSCs may have higher proliferation potential and that in effect ALP-positive cells dominate the cell culture, which could explain the observed increase of ALP activity over prolonged culture. This hypothesis can be supported with the study by Lee et al. [43] revealing the higher expression of PKCδ with higher ALP activity in human MSCs; and observations revealing the positive correlation between PKCδ expression and cell mitotic activity [47]. This hypothesis could also be supported with the previous observation of ALP overexpression in germ cell tumors [48]. Interestingly, pluripotent stem cells, including human ESCs and iPSCs, are characterized by high membrane expression of ALP that is recognized as one of the pluripotency markers. Moreover, high ALP expression is a distinctive feature of pericytes, recognized as MSC progenitors, which were shown to exert a cardioprotective effect in heart ischemic injury, and are recognized as the myogenic precursors in skeletal muscles [49–51]. Nevertheless, the analyzed E-ADSCs did not reveal overexpression of the OCT4 and Nanog genes. The achieved results point to the potentially different role of ALP activity in ADSCs, apart from its influence on the differentiation ability (osteogenesis) analyzed in vitro. This calls for further studies into ALP function in stem cells and its influence on their proliferation potential.

Interestingly, we have observed a much higher expression of the zinc finger transcription factor GATA4 in E-ADSCs as compared with other ADSC subtypes. GATA4 overexpression activates the promoters and enhancers of the alpha-myosin heavy chain and cTNT in models of cardiogenesis [40, 52, 53]. The crucial role of GATA4 in cardiac morphogenesis has been confirmed previously with embryonic lethality in GATA4 knockout mice [54]. The contribution of epicardium-derived stem cells to CMs remains a matter of debate, with a few studies confirming derivation of the CMs from proepicardial stromal cells [55–57]. The observed high constitutive expression of GATA4 in E-ADSCs may point to their high plasticity toward a cardiomyogenic lineage. Thus, we analyzed the ability for ADSCs to differentiate into CMs, applying two methods described previously. We did not observe an increase in Actn2 and cTNT expression, as well as myogenic change in cell morphology after 5-aza-induced cardiac differentiation. Our observation is consistent with data presented by Lee et al., Safwani et al., and Balana et al. [58–60], but remains in contradiction to previous findings confirming effectiveness of this method in differentiation of BM-MSCs into CMs [33, 61]. Subsequently, we applied a method described by Fu et al., who identified a minimum cocktail of seven transcription factors inducing transdifferentiation of human fibroblasts into CMs [24]. This work was preceded with an experimental study presenting successful derivation of the beating CMs from rodent fibroblasts, induced with three factors only (GATA4, Mef2c, and Tbx2), as well as a successful in-vivo reprogramming of the cardiac fibroblasts to CMs [41, 62]. A few analyses underline the fact that there is not a specific marker allowing a distinction between fibroblasts and ADSCs. Indeed, particular fibroblast subsets can meet part or all of the criteria defining MSCs. We confirm this observation, revealing the stem cell specific phenotype of the BJ fibroblasts and their osteogenic potential. Thus, MSCs and fibroblasts are hypothesized to be just different states in a single cellular family [63, 64]. Consequently, we have concluded that a technique described previously might be applicable for ADSCs, putatively with higher effectiveness, taking into account their multipotent characteristics. Our results confirm this hypothesis, with observation of the alpha-actinin-positive stem cell derivatives, overexpressing Actn2 and cTNT. Nevertheless, because single cells revealed a myogenic phenotype, we have not observed beating cells within 3 weeks of observation. Analyzing all ADSCs, the resultant differentiation towards CMs correlated positively with the constitutive GATA4 expression. This could explain the observed variation of the reprogramming effectiveness in the E-ADSC samples, unless this dependence was not observed for the selectively analyzed E-ADSCs. Because GATA4 is one of the transfected factors, we hypothesize that higher constitutive GATA4 expression in E-ADSCs might be one of many other factors responsible for their higher cardiac potential revealed with Actn2 and cTNT overexpression. It is of note that we examined the phenotype and genotype of the transfected cells. This is the limitation of our study and provides scope for further future research, including electrophysiological evaluation of E-ADSC-derived CM-like cells, as well as experimental trials on the application of E-ADSC-derived CMs in the experimental model of myocardial ischemia, with subsequent comparison with iPSC-derived CMs.

The presented results are consistent with previous observations of successful cardiac and subcutaneous fibroblast transdifferentiation, as presented by Srivastava’s team [24]. Their approach makes feasible the in-situ transformation of the postinfarction scar into a functional myocardium. Our observation of the high E-ADSC cardiomyogenic potential may allow complementary treatment based on an in-vitro ADSC differentiation into CMs with an autologous epicardial adipose tissue sample harvested through the minimally invasive technique. Despite the fact that subcutaneous fibroblasts are more easily obtainable and thus provide a more convenient source for induced CMs, our results point to the higher cardiogenic capacity of E-ADSCs, which calls for further studies into their potential role in cardiac regeneration. Taking into account the fact that epicardial fat constitutes 15 % of the heart mass and mean epicardium thickness is 5.3 ± 1.6 mm [65, 66], the alternative approach may comprise a direct stimulation of E-ADSCs, with their recruitment to the ischemic territory and in-vivo transdifferentiation into CMs. This is supported by our observation of the high proliferation abilities of E-ADSCs in vitro.

Four of five epicardial adipose tissue samples were harvested from the heart transplant recipients (explanted hearts). This group of patients was insignificantly older and burdened with comorbidities, which according to previous observations could affect negatively the multipotency of the MSCs [46]. Despite this fact, the achieved results point to the outstanding properties of E-ADSCs. Furthermore, heart transplant recipients represent the target population for the proposed therapeutic approach, which increases the clinical significance and applicability of the achieved results. Similarly, comparison of E-ADSCs and P-ADSCs harvested from the same brain-dead organ donor, free of chronic morbidities, revealed distinct characteristics to stem cells derived from epicardial adipose tissue. The fact that not all respective ADSC subsets were harvested from the same donors is a limitation of this study.

Just a few studies comparing characteristics of human ADSCs from different sources have been already reported. Baglioni et al. [67] analyzed genotypes, phenotypes, and differentiation abilities of the subcutaneous and omental ADSCs, showing no significant differences. The analysis of intrathoracic fat depots as the source of the ADSCs was the subject of just a few studies. Wang et al. compared rodent pericardial and subcutaneous ADSCs showing similar surface marker phenotype, and lower osteogenic and adipogenic potential in P-ADSCs. Concurrently, P-ADSCs showed higher GATA4 and MEF-2C constitutive expression with the presence of cTNT-positive cells after 5-aza stimulation [68]. Recently, Naftali-Shani et al. presented a comparative analysis of human epicardial (aortic root fat pad), pericardial, and subcutaneous MSCs confirming their similar cellular phenotype. Interestingly, the authors observed a higher cardioprotective effect of human subcutaneous ADSC transplantation, as compared with E-ADSCs, in the rodent model of MI. These unexpected results were explained by a lower anti-inflammatory characteristic of E-ADSCs as compared with P-ADSCs and subcutaneous stem cells [69]. This remains in accordance with previous observations pointing to the MSC-mediated improvement of postischemic heart function predominantly through the paracrine effect [70]. Nevertheless, these data do not exclude a potential application of E-ADSCs in cardiac regeneration through effective in-vitro cardiogenesis, based on their unique cardiogenic capacity.

## Conclusions

The presented data point to a significant dissimilarity in reprogramming potential of different human ADSC subsets. Human E-ADSCs are characterized by higher cardiomyogenic potential as compared with the other ADSC subtypes. This observation points us to E-ADSCs as a valuable subject for further studies into a more effective method of MSC differentiation into CMs.

The presented results call for further studies into the role of ALP and its influence on ADSC proliferation and differentiation abilities. Taking into account the high constitutive ALP expression, application of ALP as the marker of successful ADSC osteogenic differentiation should be always accompanied by the respective control.

## Abbreviations

5-aza, 5-azacytidine; Actn2, alpha-actinin 2; ADSC, adipose-derived stem cell; ALP, alkaline phosphatase; BM-MSC, bone marrow-derived mesenchymal stem cell; CM, cardiomyocyte; CPC, cardiac progenitor cell; cTNT, cardiac troponin T; DMEM, Dulbecco’s modified Eagle medium; E-ADSC, epicardial adipose-derived stem cell; ESC, embryonic stem cell; FBS, fetal bovine serum; iPSC, induced pluripotent stem cell; IRB, institutional review board; MI, myocardial infarction; MSC, mesenchymal stem cell; O-ADSC, omental adipose-derived stem cell; P-ADSC, pericardial adipose-derived stem cell; PBS, phosphate-buffered saline
